# The Crick-Eatery: A Novel Approach to Evaluate Cricket (*Acheta domesticus*) Powder Replacement in Food Products through Product Eating Experience and Emotional Response

**DOI:** 10.3390/foods11244115

**Published:** 2022-12-19

**Authors:** Isaac Ho, Adelynn Peterson, Jack Madden, Kylie Wai, Ruta Lesniauskas, Jeff Garza, Attila Gere, Samir Amin, Amy Lammert

**Affiliations:** 1 Department of Food Science and Nutrition, California Polytechnic State University, San Luis Obispo, CA 93407, USA; 2 Garza Consulting, Evanston, IL 60201, USA; 3 Garza Consulting, Grand Rapids, MI 49501, USA; 4 Institute of Food Science and Technology, Hungarian University of Agriculture and Life Sciences, H-1118 Budapest, Hungary

**Keywords:** cricket powder, sausage, pasta, chocolate brownies, plate waste, emotions, subjective satiety, acceptability, three-course meal

## Abstract

This study was conducted to evaluate three different food products containing cricket powder for consumer acceptability, emotional response, satiety, and plate waste. US untrained consumers (*n* = 108), from the San Luis Obispo, CA area, were recruited to evaluate three food products (sausage, pasta, and brownies) as components in a three-course meal that either contain cricket powder (CP) or not (Control). The CP sausage was found to have lower liking scores than the Control for the attributes tested (*p* < 0.05). The CP pasta was found to be higher in overall liking than the Control (*p* < 0.05). The CP Brownies were rated highly across the attributes, except for texture and aftertaste (*p* < 0.05). Though the CP products were found to be as acceptable as the Controls, the use of cricket powder may have affected the texture and flavor profile of both the CP sausage and brownies. The participants selected more positive emotions terms for both the CP and Control products than negative emotions. Negative terms selected, such as worried, decreased once the products were consumed (*p* < 0.05). Plate waste and subjective satiety may also be indicators of consumer acceptability. Significant correlations were found between appearance liking and satiety as well as taste liking and plate waste for both the Control and CP products/dishes (*p* < 0.05). Based on this work, future acceptance of insect-based products may be encouraged by evaluating the products throughout an eating experience.

## 1. Introduction

By 2050, the human population is expected to reach 9 billion [1]. To feed the growing population, the United Nations found that food production will need to double, including protein [2]. The global protein market was found to be worth 38 billion USD in 2019 and is expected to grow at a rate of 9.1% from 2020 to 2027 [3]. Currently, the protein market is mainly comprised of animal protein production, which continues to grow as developing countries adopt Western practices such as meat consumption [4]. However, traditional animal protein production, or livestock farming, contributes to the overconsumption of limited natural resources, such as land and water, and the production of greenhouse gas emissions [4,5]. As a result, sustainable methods may need to develop to mitigate the effects of livestock farming, but also provide protein-rich food sources.

Alternative protein sources, such as plant-based proteins and cultured meat, have been developed for commercial sale and may offset the demand for animal proteins [3]. However, when replacing or reducing animal protein consumption, not all protein alternatives may meet every consumer need. Plant-based proteins may not be as comparable to animal proteins due to food functionalities, which may affect the sensorial aspects of plant-based products [6,7]. Cultured meat, or lab-grown meat, may deter consumers due to its “unnatural” perception by some, but also its higher cost of production than conventional animal meat may reduce its accessibility to some consumers [8,9]. Therefore, a variety of alternative proteins may need to be developed, as certain consumer groups may value one alternative over another [10].

Entomophagy, the practice of consuming insects, may be one of those alternatives. Insect consumption has historically, and continues to be, a food source for two billion people across countries in Asia, Latin America, and Africa [4,11,12,13,14]. For regions where livestock farming may be limited, insects may be a viable option. Compared to conventional animal protein, insects can provide all the essential amino acids necessary for human nutrition but require fewer resources, such as lower feed and land requirements, than livestock [5,15,16,17]. Therefore, insects may offer new opportunities in the food industry to develop protein-based products.

Currently, there has been investment in producing insects for human consumption. There are over 2000 edible insect species that are consumed across certain regions around the world [18]. However, selecting which insect species to farm may need to be considered, as each species may vary in nutritional profile [16,19]. In China, 34 edible insect species, which include silkworm larvae (*Bombyx mori*), yellow mealworm (*Tenebrio molitor*), and Orthoptera species (locusts, grasshoppers, and mole crickets), have been developed and assessed for production for human consumption [20]. In Western countries, there has been a focus on developing methods to farm the house cricket (*Acheta domesticus*) and yellow mealworms for human consumption [18,21]. Western governments, such as the EU, have even started to recognize and regulate insect species, such as yellow mealworms, as food for humans [22].

Insect production may raise concerns, as it is a new and developing method of food production. High levels of pathogens or other contaminants, such as heavy metals found in the feed used in insect farming, may be the main vector of contamination for insects intended for human consumption [4,23]. Not to mention, insects may be considered an allergen concern. Insects have a similar protein profile to shellfish/crustaceans that may cause allergic responses in certain individuals [24,25]. However, these concerns may be managed through regulations on agricultural feed, and preventative controls such as HACCP procedures and required labeling on insect-based foods [4,23]. Requirements for high-quality feed for insects intended for human food may lead to higher production costs when compared to conventional animal meat production [26]. As a result, investment into developing insect farming may be necessary to increase the productivity and profitability of the insect market. In Europe, nearly 90% of the investment costs of insect farming are covered by venture capital firms in Europe [21]. In North America, startup companies, such as Aspire Food Group and All Things Bugs (GrioPro^®^), have been funded by both government and private bodies for cricket farming, for both the food and agriculture industries [18]. However, when introducing insects into the Western market, insects may not be as accepted as a food source.

The future acceptance of Insects as food may be influenced by psychological factors that may deter consumers from eating them. Disgust, a culturally induced form of rejection, is a way for humans to avoid foods that may cause illness and disease [27,28]. In Western culture, insects are considered vectors of disease as some consumers view them as filthy or associate them with contamination and spoilage [27,29,30,31]. Food neophobia, the avoidance of trying new, unfamiliar foods, may also influence consumers with the intention to consume insects [32,33]. Lombardi et al. [34] found that food neophobia negatively affected the willingness to pay for insect-based products among Italian consumers. For Western consumers, insects may be considered a novel food as conventional livestock are considered the predominant protein source. This may be the result of insects in Western regions being smaller than insects found in tropical regions, which may have led to insects in Western regions being seen as an insubstantial food source [35]. As a result, both factors may affect influence the future adoption of insects by Western consumers [36].

Attitudes towards insects may also predict the willingness/intent to consume insects [33,37,38]. La Barbera et al. [31] found that positive attitudes (low disgust and high interest) towards insects, as well as individuals’ openness to new food experiences among Danish and Italian consumers, may encourage the consumption of insects. In China, consumers who have previously consumed insects have been found to have positive attitudes towards them, with less self-reported disgust, and would be more willing to eat them than those who have not [39,40]. In Latin American countries, regions that do not typically incorporate insects into their food may be unfamiliar with the practice; however, consumers have been found to be more willing to eat insects if prepared in familiar ways, such as being fried or roasted [41,42,43]. To better predict the acceptance of insects, methods to change the attitudes of consumers and increase their familiarity with entomophagy may help to move past the barriers of disgust.

Previous research has focused on identifying interest to consume insects, which may increase their acceptance. One strategy may be to identify early adopters, the first group of consumers who gain interest and accept a new product [44,45]. Once accepted by this group, widespread adoption may occur within the market [46,47,48,49]. Another way may be to inform consumers of the sustainable benefits of consuming insects. The addition of information regarding entomophagy, when evaluating insect-based products, has been found to either increase interest/intent to consume insects or increase positive emotions during testing [34,47,50,51,52]. Serpico et al. [50] suggested that increasing positive emotions, such as curiosity and interest, while decreasing negative emotions, such as disgust and worry, may encourage consumers to eat insects. As a result, identifying consumers with a high interest in insect-based products may be a viable way to introduce insects as a food source.

To increase interest to consume insects, determining the delivery method, or the product appropriateness, may be a consideration. Product appropriateness, or the perception of what is deemed acceptable by consumers, such as when incorporating an ingredient like insects, may increase acceptance by consumers [53,54,55]. When incorporating insects into food products, nonvisible forms, such as powders and ground meals, may be a viable option. Western consumers may be more willing to try insect-based products if they contained nonvisible insects than visible forms (whole insects) [30,33,56,57]. Cricket powder or powders from other Orthoptera species may be a viable insect powder for Western consumers as it has been found to contain a high protein content (42.0–65.5% dry matter) [58,59,60]. Not to mention, products that contain cricket powder are available in the Western market, including protein bars (EXO), pasta (Bugsolutely), and snack foods (Chirps) [61]. However, when incorporating insects into conventional food products, consumers may have certain expectations for the sensory characteristics of these products. Cricket powder has been found to decrease overall consumer acceptability, such as in terms of textural properties, of products and may lead to rejection by consumers [52,62].

When developing food products, especially novel foods such as insects, predicting their future acceptance may go beyond consumer liking when insect-based products are compared to their conventional counterparts. In a preliminary study, Ho et al. [63] developed three different food products containing cricket powder: sausage, pasta, and chocolate brownies. These products were evaluated for nutritional content and quality parameters. To further determine if these products are comparable to conventional food products, understanding how consumers may eat these products in normal eating situations may be a consideration. Previous studies have focused on the food-eating experience and emotions as predictors of consumer acceptance and food-choice behavior [64,65,66,67]. Consumer liking and positive emotions were found to increase when consumers deemed food products appropriate [66,67,68]. Insects, which may invoke negative emotions such as disgust by consumers, may benefit from an evaluation of their products under these conditions, by increasing more positive emotions [68]. Not to mention, there is limited research on complete dishes involving insects, as prior studies have evaluated mainly snack foods (chips, crackers, cookies, etc.), which is a method of easily incorporating insects into food products [33,62,69,70].

As a result, the three food products (sausage, pasta, and brownies) were developed into a three-course meal that US consumers would evaluate. This study was conducted (1) to evaluate different product forms containing insect powders, (2) to understand and identify psychological factors and attitudes that may influence the consumption of insects among US consumers, (3) to evaluate consumer acceptability of insect-based foods with emotional response, and (4) to determine if the product eating experience (food delivery method, plate waste, and satiety) affects the future acceptance of insects as food.

## 2. Materials and Methods

**Figure 1:**
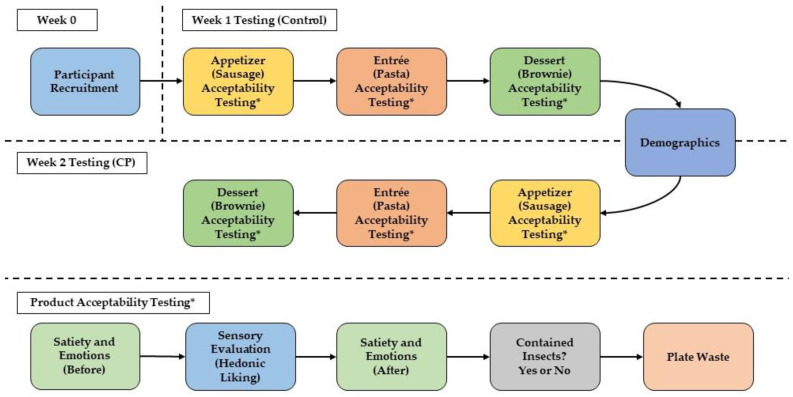
Flow diagram of the sensory test conducted. The Product Acceptability Testing flow diagram describes the factors measured during each product evaluation. * For week 1 and week 2 of acceptability testing for all products evaluated, subjects answered four questionnaires, subjective satiety and emotions before and after consumption, hedonic questionnaire, and answered whether or not the product contained insects. Upon completion of testing, all products were weighed to determine plate waste.

A two-week sensory test was conducted to evaluate the three different products containing cricket (*Acheta domesticus*) powder. A mild Italian-style fresh sausage, dried pasta, and chocolate brownie were developed to either NOT contain cricket powder (Control) or contain cricket powder (CP) by Ho et al. [63]. The three products were evaluated by utilizing them as major components in a three-course meal that included an appetizer (sausage), entrée (pasta), and dessert (brownie). The CP sausage, pasta, and brownies were developed to contain optimum usage levels of cricket powder (6.23%, 5% and 7%, respectively), to maximize cricket powder replacement without affecting quality parameters [63]. Participants were asked to evaluate the products used in their respective dishes. Figure 1 describes how the sensory test was conducted and the variables (consumer acceptability, emotions, subjective satiety, and plate waste) that were measured throughout the test. All online questionnaires for recruitment and sensory testing were developed and completed using Red Jade sensory software (Martinez, CA, USA). During Week 1, participants were asked to evaluate the three-course meal containing Control products while Week 2 had CP products. Participants were pre-screened to be willing to consume products containing insects over the course of the study; however, for the purpose of this study, they were not informed which products either contained insects or not during each week. Each week was conducted as blind taste testing, where the participants were asked to evaluate each dish without being given information regarding whether the products contained insects or not. After the completion of both weeks of testing, the participants were informed which dishes from each week contained insects (CP) or not (Control). Previous research, which is further explained later, has found that this may affect how consumers perceive the product, which can affect evaluation [51,71].

### 2.1. Products Tested and Sample Preparation

**Figure A1:**
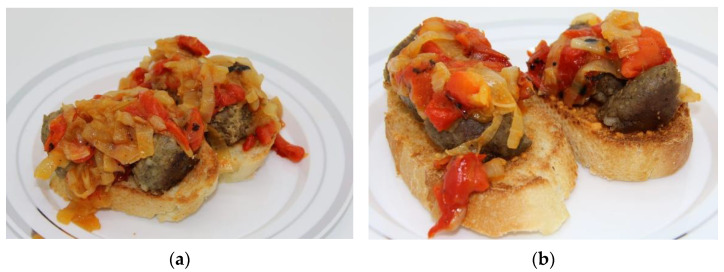
Sausage bruschetta appetizer presented to participants for product evaluation. (**a**) Sausage bruschetta contained the Control sausage used in the study and (**b**) the sausage bruschetta contained the CP sausage.

**Figure A2:**
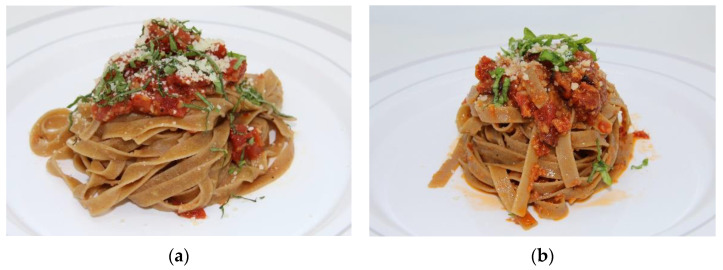
Pasta entrée presented to participants for product evaluation. (**a**) Pasta dish containing the Control pasta used in the study and (**b**) the pasta dish containing the CP pasta.

**Figure A3:**
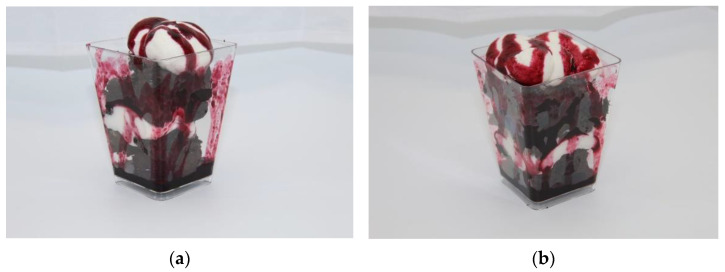
Brownie dessert that was presented to participants for product evaluation. (**a**) Brownie dessert contained the Control brownie used in the study and (**b**) the brownie dessert contained the CP pasta.

The products were incorporated into dishes for the three-course meal described in Appendix A. The formulations for both the Control and CP versions of the sausage, pasta, and brownies, and how they were prepared, are described by Ho et al. [63]. The same cricket powder (JR Unique Foods, Thani, Thailand) used in the previous study was used for this study [72]. The three dishes used for the three-course meal were the following: a sausage bruschetta with an onion and pepper topping (Figure A1), pasta with charred-tomato sauce (Figure A2), and brownie trifle dessert with berry sauce (Figure A3). Products and dish components that were not made the day of were made exactly one week before each testing day. All ingredients were purchased from local suppliers/grocery stores within the San Luis Obispo, CA area and stored properly before use.

Since plate waste, later discussed, was a factor in this study, each participant was given substantial amounts of each product/dish that consumers may typically be served in food-eating situations. Serving sizes were determined by increasing the reference weights (g), described by the FDA guidelines for the Reference Amounts Customarily Consumed (RACC) [73], for appetizers, dried pasta, and brownies by 20%. The increased serving sizes based on the RACC for the sausage appetizer, dried pasta, and brownies were targeted to be approximately 105 g, 66 g, and 48 g, respectively. The RACC for dried pasta and brownies was selected as there is no reference amount, in weight, for an entrée or dessert. However, components, aside from the main product components for each dish, were measured to limit differences in weight when served to participants. The average weights of the appetizer, entrée, and dessert dishes (excluding the weight of the serveware) were 119.1 g, 201.3 g, and 78.3 g, respectively.

Products were prepared and served in a manner similar to how consumers may eat them in normal dining situations (Figure A1, Figure A2 and Figure A3). Dishes were prepared on premium disposable plates (Munfix, Mezlex Corp, Monroe, NY, USA) and dessert cups (Eupako, Eupako Co., Ltd., Shenzhen, China) for presentation purposes.

#### 2.1.1. Appetizer Preparation

The appetizer components were developed using the formulations found in Table A1. Prior to testing day, the sausage was prepared and weighed into 40 g patties and vacuum-packed in bags. The sausage patties were placed into a walk-in freezer set at −26.1 °C.

During the day of testing, for the onions and pepper topping, the olive oil and sliced onions were cooked over a stovetop until the onions were caramelized. The roasted red peppers were sliced and added to the caramelized onions and mixed until homogenous. The topping was kept in a hot water bath at 60 °C before serving.

For dish preparation, the vacuum-packed sausages were placed into a hot water bath set at 71.1 °C, using an Anova Precision Cooker (Anova Culinary, San Francisco, CA, USA), for at least 1 h before preparation. The bread was sliced at an angle to be about 1.5 cm in thickness. The bread slices were topped with approximately 1 g of parmesan cheese on each slice and toasted in a kitchen oven using the low broiler setting for 5 min or until the top was golden brown in color. The cooked sausages were removed from the hot water bath and placed into an Avantco sandwich grill (Model P70S, Meridian, ID, USA) set at 176.7 °C (350°F) for 1 ½ min to sear. The sausages were removed from the grill and each patty was sliced into 6 pieces. To assemble, two slices of toasted bread were placed on a plate, followed by one sausage patty (3 pieces per slice), and 59.1 mL (using a ¼ US measuring cup) of the onions and peppers topping was placed over it. The dish was served immediately.

#### 2.1.2. Entrée Preparation

Entrée components were prepared using the formulations found in Table A2. Prior to testing day, the pasta was prepared and allowed to dry. The dried pasta was pre-weighed to be approximately 66 g and placed into individual plastic bags for storage. For the tomato sauce, the canned diced tomatoes, garlic, salt, pepper, and the first addition of olive oil were mixed in a large container until homogenous. The tomato mixture was poured into baking sheets lined with aluminum foil prior to baking. The sheets were placed into a Combi Oven (Rational Model CPC 102, Landsberg am Lech, Germany) set at 218.3 °C (425°F) for 30 min, or until the tops of the tomatoes were charred. The sheets were removed from the oven, and the sauce was poured into a large container. The second addition of olive oil was added to this and mixed thoroughly, and the tomato sauce was allowed to cool at ambient temperature until ready to handle. The tomato sauce was sealed in vacuum pack bags and placed into a walk-in freezer set at −26.1 °C prior to testing day.

During sensory testing, the tomato sauce bags were placed into a hot water bath using an Anova Precision Cooker (Anova Culinary, San Francisco, CA, USA) set at 73.9 °C (165°F) for at least 1 h prior to serving. The tomato sauce was then poured into hotel pans that were in a hot water bath set at 73.9 °C (165°F). The pasta was boiled for 4 min, drained, and placed into mixing bowls. Then 59.1 mL (1/4 US measuring cup) of tomato sauce, using a 2-ounce (59.1 mL) ladle, was poured onto the pasta and mixed using a pair of tongs. The dressed pasta was immediately placed onto a plate and topped with the residual tomato sauce from the bowl, 1 g of parmesan cheese, and 1 g of fresh basil. The pasta was served immediately.

#### 2.1.3. Dessert Preparation

Formulations for each dessert component can be found in Table A3. Prior to testing, the brownies were prepared and baked in 18 in by 13 in (45.7 cm by 33.0 cm) sheet pans in a Combi Oven set at 162.8 °C (325°F) for 25 min. The brownies were removed from the oven and allowed to equilibrate to room temperature before storage. The brownies were wrapped in aluminum foil and placed in a walk-in freezer set at −26.1 °C. For the berry sauce, the frozen berries, sugar, and water were mixed in a pot and heated over a stovetop. The berry mixture was allowed to be heated until the sauce reached a Brix of 40 using a refractometer (Atago Model 3810, Atago USA, Inc., Bellevue, WA, USA). The sauce was immediately removed from the stovetop and strained to remove the pulp and seeds. The strained sauce was poured into a 32 oz deli container and placed into a walk-in freezer for storage. The berry sauce was placed into a fridge set at 4 °C a day before testing.

During testing, the brownies were removed from the freezer and allowed to equilibrate to room temperature for at least 2 h. The brownies were cut into 1.5 cm by 1.5 cm squares (brownie bites) for preparation. Heavy cream, sugar, and vanilla extract were mixed using a stand mixer (Model KitchenAid Pro 600 KP26M1X, KitchenAid, Benton Harbor, MI, USA) for the whipped topping. The mixture was whipped until stiff peaks were formed. The whipped topping was poured into Ziploc resealable bags that would be used to pipe the topping onto the dessert.

For assembly, the brownie bites were pre-weighed to be 48 g for each serving. For the berry sauce, 8 mL of sauce was measured using a 10 mL syringe (Care Touch, Westminster, CO, USA) for each serving. Half of the berry sauce (4 mL) was used to line the inside of the dessert cup followed by half of the 48 g of brownie bites. About 1 US tablespoon (14.8 mL) of whipped topping was piped onto the brownie bites followed by the rest of the brownie bites. Another 1 tablespoon of whipped topping was piped onto the second layer of brownies and the remaining 4 mL of berry sauce was drizzled over the whipped topping. The brownie dessert cups were placed into the refrigerator and/or served immediately when needed.

### 2.2. Ethics Statement

This research was reviewed and approved by the Cal Poly San Luis Obispo Institutional Review Board (2022-037-CP).

### 2.3. Participants and Recruitment

A recruitment email was sent through the Cal Poly SLO Sensory consumer database in Red Jade to untrained consumers. The email contained a website link to answer a screener questionnaire. Consumers would be qualified for participation if they had the following: age 18 and over, no declared food allergies and/or intolerances, had a consumption frequency of at least once a month for two of the three products tested (sausage, pasta, and brownies), be willing to consume food products containing insects and non-visible insect powders, and able to come in during the days and times offered for the sensory test. After screening, 164 people were qualified for the 2-week sensory test and were sent a follow-up email with instructions on how to participate. All 164 qualified consumers were recruited, expecting absences over the course of study. Testing occurred on Monday and Wednesday from 1:30 pm to 4:30 pm PST, due to facility availabilities, with six 30-min sessions with 10 people per session. There was a one-week gap between the weeks of testing to act as a washout period for the participants. Qualified participants were informed that the sensory evaluation would involve consuming a three-course meal and were asked to sign up for at least one session offered either Monday or Wednesday each week. Participants would receive a $25 Amazon gift card after the completion of each week. By the end, 108 subjects (*n* = 108) were used in the study, with 106 (*n* = 106) of the 108 participating in Week 1 (Control) and 103 of the 108 (*n* = 103) participating in Week 2 (CP). Overall, 102 of the 108 subjects (*n* = 102) participated in both weeks of testing.

### 2.4. Data Collection and Study Design

#### 2.4.1. Testing Room Setting and Procedure

The three-course meal was prepared in the culinary lab kitchen, ready-to-serve, and given to participants who were waiting in the classroom attached to the culinary lab at Cal Poly, San Luis Obispo (Cal Poly SLO). Due to the facilities at Cal Poly SLO, there is a teaching classroom adjacent to the culinary kitchen. Testing was conducted there for food preparation reasons to ensure that the optimum temperatures of the products/dishes were similar to how consumers may be served in a restaurant setting and/or normal eating situations. Though this study did not focus on a restaurant setting, the products were served restaurant-style due to the variables, later explained, that were being measured. The tables in the classroom were arranged at the perimeter of the room so that participants would be facing away from each other during testing. The tables were lined with white plastic table covers (Prestee, Perch HQ, Boston, MA, USA) and participants were given silver-lined plastic silverware (Munfix, Mezlex Corp, Monroe, NY, USA) to eat with. To simulate a food-eating situation when consuming a three-course meal, no saltine crackers were provided over the course of the study and the participants were instructed to drink the water provided between each dish.

When participants checked in for their session, they were given their participant number attached to a table card holder (Hohiya, Hohiya Enterprise Co., Ltd., New Taipei City, Taiwan), which is typically used at some restaurants to indicate their order number. Participants were asked to be seated with only one person per table and with at least one empty seat between each other. Participants were given iPads with iOS 15.4.1 software (Model MP2F2LL/A, Apple, Cupertino, CA, USA) which gave them access to Red Jade website to answer questions for product evaluation. Before testing, participants were informed that they would be evaluating a three-course and that they could eat as much as they wanted of each dish. To indicate that they had finished eating each dish, they were asked to move the cardholder with their participant number from the left to the right side of their table. This would indicate that they were finished eating, and that their plate could be removed from their table and replaced with the next dish item. After all product evaluation was completed and they had finished consuming the last item (dessert), they were allowed to leave the room and had completed that week of testing.

#### 2.4.2. Consumer Acceptability Testing

After being seated and ready to begin, participants were first given the sausage bruschetta appetizer, followed by the pasta entrée, and lastly, the brownie dessert. Each dish was labeled with a 4-digit code that participants had to enter into the Red Jade questionnaire; this would lead them to the next questionnaire page that contained a set of questions pertaining to the dish/product. Once the sample code was entered, participants responded to the questions that evaluated the products for the following attributes on a 9-point hedonic scale (1 = Dislike Extremely to 9 = Like Extremely): appearance, aroma, overall liking, taste, texture, flavor, and aftertaste.

Due to the sausage bruschetta appetizer being a more complex dish than the pasta entrée and brownie dessert, consumers evaluated the sausage and the whole bruschetta for the attributes tested. Consumers only evaluated the pasta product and brownie product from their respective dishes.

After evaluating each product/dish, participants were asked on a binomial scale if the dish contained insects (1 = Yes) or did not contain insects (0 = No). This was to determine if the participants were able to discern which products contained insects over the course of the study.

#### 2.4.3. Emotional Response

Emotional responses were evaluated before and after consuming each product/dish. A modified version of the EsSense25 questionnaire [74], a check-all-that-apply (CATA) list of emotion terms, developed by Serpico et al. [50], was used to evaluate the emotions participants felt. The list of emotion terms was selected to reduce panelist fatigue when answering all of the questions. The original EsSense25 questionnaire includes a list of 25 emotion terms. The modified version used in the study included a 26th emotion term, “daring”, from the EsSense Profile^®^ [75], which may be associated with the interest to consume insects [50]. When answering the CATA, participants were asked to select any emotion terms they were feeling during evaluation. If the participants were to select the term “disgusted”, the questionnaire would ask them a follow-up question to rate their feeling of disgust (Disgust Rating) [50] on a 5-point scale (1 = Not Disgusted at All; 5 = Extremely Disgusted). Participants were asked to answer the CATA questions before and after the hedonic questions when consuming each of the three dishes tested.

#### 2.4.4. Plate Waste and Satiety

Since the products were served at amounts consumers may typically consume them, plate waste was measured over the course of the study. Before a dish was served to a participant, the dish was labeled with the participant’s code, hidden from view, to keep track of which plate belonged to which participant. The dish was weighed as the initial weight using a digital balance scale (Model Adventurer Pro AV3102, Ohaus Corporation, Parsippany, NJ, USA) before being served to the participants. Scales were calibrated with a 1 kg and 2 kg standard stainless-steel weight (Models S-20879 and S-15293, Uline, Pleasant Prairie, WI, USA) before each of testing. Once the participants indicated they had finished done consuming the dish, it was removed and weighed as the final weight. Plate waste was determined using the following equation:

$$\mathrm{Plate}\;\mathrm{Waste}\;(\%)=\frac{\mathrm{Final}\;\mathrm{Weight}\;\mathrm{of}\;\mathrm{Dish}-\mathrm{Average}\;\mathrm{Plate}\;\mathrm{Weight}}{\mathrm{Initial}\;\mathrm{Weight}\;\mathrm{of}\;\mathrm{Dish}-\mathrm{Average}\;\mathrm{Plate}\;\mathrm{Weight}}\times 100\%$$

where, the average plate weight was determined by weighing 10 plates used for each respective dish. This was performed in triplicate.

Another factor to consider may be the level of satiety participants may have experienced before testing and after consuming each dish. Participants rated their subjective satiety on the Satiety Labeled Intensity Magnitude (SLIM) scale [76], which rated from 0 = Greatest Imaginable Hunger to 100 = Greatest Imaginable Fullness. Participants were asked to rate their satiety before and after the sensory evaluation/consumption of each product/dish.

#### 2.4.5. Sociodemographics, Attitudes and Beliefs towards Entomophagy, Food Neophobia

To limit the fatigue participants may have had while evaluating the products, a website link to access the demographics questionnaire was emailed through Red Jade after the first week of testing. Participants were asked to answer sociodemographic questions which included the following: age group, gender, ethnic background, highest level of education, diet with respect to animal products, and insect consumption familiarity.

To evaluate the participants used in this study and their attitudes toward entomophagy, they were asked to answer the Entomophagy Attitudes Questionnaire (EAQ) [41]. The EAQ evaluated three factors that included five statements regarding the disgust towards insects (EAQ-D), three statements about the interest to consume insects (EAQ-I), and two statements regarding the use of insects as animal feed (EAQ-F). Participants were asked to rate their agreement with these statements on a 7-point scale (1 = Completely disagree; 7 = Completely agree). To the authors’ knowledge, the EAQ has not been used to evaluate US consumers [39,41]. This study would also be the first to evaluate the attitudes of consumers who would be consuming insect-based products.

The demographics questionnaire included other questions regarding the consumption of insects. A set of questions developed by Rovai et al. [47] was used to determine which cluster groups (No-thank-yous, Hideaways, Daredevils, and Peekaboos) the participants aligned towards. The set of questions included questions that evaluated the participants’ beliefs about consuming insects, lifestyle, and purchasing of food on a 5-point agreement scale (1 = Disagree completely; 5 = Agree completely), and liking of products containing nonvisible insects on a 5-point Likert scale (1 = Dislike very much; 5 = Like very much). Previous research using this set of questions was conducted through online surveys that evaluated the concept of insects as food [46,47]. Therefore, this study wanted to evaluate consumers who were willing to consume insect-based products in person.

Another factor that was measured was food neophobia. Participants were asked to answer the Food Neophobia Scale (FNS) [77] by rating their agreement with ten statements on a 7-point scale (1 = Completely disagree; 7 = Completely agree). By the end of the study, 106 subjects (*n* = 106) of the 108 responded to the demographics questionnaire.

### 2.5. Data Analysis

Analyses were conducted using R-project (version 4.1.3, R Foundation, Vienna, Austria) and RStudio (Version 2022.07.2, RStudio, PBC, Boston, MA, USA) to compare differences between the Control and CP product/dishes [78]. All responses collected as binary responses, Yes/No if it contained insects or emotional responses, were rescaled as percentages of participant selection to better analyze the results. The multcomp, multcompView, emmeans R packages and the aov function were used for means being compared, using a two-way ANOVA. The multcomp and multcompView R packages and the prop.test function were used to compare differences in percentages using a two-tailed *z*-test. A 95% confidence level (α = 0.05) was used for all statistical analyses.

#### 2.5.1. Sociodemographics, Attitudes and Beliefs towards Entomophagy, Food Neophobia

The proportions and means for the sociodemographics, EAQ, and FNS were determined based on the participants’ responses. Scores of the food neophilia statements from the FNS were reversed [32,77]. Participants were assigned to a cluster group (No-Thank-Yous, Hideaways, Daredevils, Peek-a-boos, or Flat) using a typing tool that evaluated the responses to the set of questions that were developed by Rovai et al. [47]. Participants who were not able to be determined to be part of any cluster group were assigned as “Flat”. From the cluster assignment, the Hideaways group (57.5%) was found to be the most predominant among the participants used for this study. The mean scores regarding the Hideaways’ attitudes and beliefs towards entomophagy and food neophobia were also determined.

#### 2.5.2. Consumer Acceptability, Plate Waste, and Insect Identification

The mean hedonic liking scores and plate waste were analyzed using a two-way ANOVA blocked on Treatment (Control and CP) and Participant. A two-tailed z-test was also conducted on the percentages of participants who answered “Yes” to whether the product/dish contained insects or did not contain insects between the Control and CP versions. For this study, participants were not informed in the beginning which dishes contained insects. All dishes in Week 2 contained insects (CP). Participants who were able to discern that the Week 2 dishes (CP) contained insects and selected “Yes” were identified as the Insect ID subgroup for each dish. Liking scores and plate waste were analyzed for the subgroup using the tests mentioned.

#### 2.5.3. Emotional Responses

Percentages of emotion term selection were determined both before and after consumption of each product/dish. Emotional responses before and after consumption were compared for differences in selection using two-tailed *z*-tests. Analyses were conducted independently for each week (Control and CP).

The change in emotion term selection (ΔEmotion) was also determined by subtracting the percentage after by the before emotion term selection (After %–Before %) for each participant. The ΔEmotion was analyzed using a two-way ANOVA, blocked on Treatment (Control and CP) and Participant, by evaluating differences in change in emotion between the Control and CP product/dishes. Analysis was also conducted to compare changes in emotion across the three-course meal (Appetizer, Entrée, and Dessert) using a two-way ANOVA, blocked on Product (sausage, pasta, and brownie) and Participant. This was followed by Tukey’s means comparisons tests to compare changes in emotions between the three different dishes.

#### 2.5.4. Relationship between Consumer Acceptability, Plate Waste, and Satiety

Mean subjective satiety was analyzed per product/dish to determine changes in satiety before and after the consumption of each dish. Analyses were conducted independently on each dish and each week (Control and CP) using a two-way ANOVA blocked on Product Evaluation (Product Before and After) and Participant. 

Pearson’s *r* correlation coefficients were determined to assess a relationship between liking scores, subjective satiety, and plate waste using the Analysis ToolPak from Excel for Microsoft 365 MSO (Version 2210 Build 16.0.15726.20188 Microsoft Corp., Redmond, WA, USA). Correlations were ran using the participant-level data as aggregate scores. Correlation coefficients were determined between the liking scores of the bruschetta, sausage, pasta, and brownies and the following: satiety before consumption, satiety after consumption, ΔSatiety, and plate waste. For each participant, ΔSatiety was determined by subtracting the subjective satiety after score by the subject satiety before score (Satiety After–Satiety Before) for each product/dish evaluated. Satiety variables and plate waste collected from the appetizer were used as aggregate scores for both the bruschetta and sausage attribute liking scores.

Correlation coefficients were determined to be significant (*p* < 0.05) using a table of critical values for Pearson’s *r* correlations using an α = 0.05 [79]. Degrees of freedom were determined by subtracting the number of pairings (*n*) by 2. For the Control and CP independent correlations, there were 4 pairings (*n* = 4) for the bruschetta, sausage, pasta, and brownie, and attribute liking scores to satiety before consumption, satiety after consumption, ΔSatiety, and plate waste. For the Control and CP (combined) correlations there were 8 pairings (*n* = 8).

## 3. Results and Discussion

### 3.1. Sociodemographics

Table 1 displays the sociodemographic information of the 106 of the 108 participants (*n* = 106) who responded to the demographics questionnaire. It also shows the participants’ familiarity with consuming insects, as well as which cluster group they were assigned to. There was a higher proportion of female participants (*n* = 72; 67.9%) than male participants (*n* = 33; 31.1%). The 35–44 Age Group was the most predominant (28.3%). The educational background varied among the participants with 77.4% having a bachelor’s degree or higher. This may be expected as recruitment was conducted through the university and the local community. The participants were familiar with the consumption of insects with 84.0% stating their familiarity with it. From the cluster group assignments, the Hideaways were found to be the most predominant (57.5%), followed by the Peekaboos group (11.3%), then the Daredevils (10.4%), and lastly the No-Thank-Yous (6.6%). The Hideaways group had the highest proportion, which may be an indicator that the participants recruited may be willing to consume insects in nonvisible forms [47]. This also supports the findings from Rovai et al. [47], that the No-Thank-Yous may reject occasions to consume insects, due to the low representation of this group, since a requirement during recruitment was the willingness to eat visible and nonvisible insects.

### 3.2. Attitudes and Beliefs towards Insects and Food Neophobia

Table 2 shows the mean scores for the EAQ factors, food neophobia scale, lifestyle assessment, purchasing of food considerations, and liking of products containing nonvisible insects of all participants, as well as the subscores for the Hideaways.

#### 3.2.1. Entomophagy Attitudes Questionnaire and Food Neophobia

For the EAQ scores, the participants self-reported, on a 7-point scale, low disgust (mean scores of 1.80–2.79 for EAQ-D statements), high interest to consume insects (mean scores of 5.75–6.14 for EAQ-I statements), and high interest to use insects as animal feed (mean scores of 5.47 and 6.14 for EAQ-F statements). When compared to the Hideaways’ subscores, the mean scores for each statement were comparable. The largest difference of 0.24 was found for the “Thinking about the flavor that a bug might have sickens me (EAQ-D)” agreement score between all participants and the Hideaways. For the FNS scores, all participants and the Hideaways subgroup self-reported low food neophobia with the highest rated statement being “I am very particular about the foods I will eat (reversed)” at 3.03 and 3.21 (on a 7-point scale), respectively.

Disgust, food neophobia, and interest/intention toward insects may play key roles in the future acceptance of insect-based foods in the Western market. Both disgust and food neophobia have been heavily studied as negative predictors of the intention to consume insects [33,36,37,70,80]. In Western countries, disgust and/or food neophobia may lead to an aversion to consuming insects. To reduce the negative perception of consumers, exposure and familiarization to the consumption of insects may increase interest. Previous studies have found that providing information about the benefits of insects or consumers tasting insects for the first time may increase the intention and/or willingness to consume them in the future [47,50,52,56,57,81,82,83]. Evaluating these three factors may provide a scope of how certain consumer groups may perceive insect-based products once introduced into the market.

The EAQ was used in this study as it has been validated to relate disgust, food neophobia, and interest across consumer groups [31]. Verneau et al. [39] had previously implemented the instrument in China. They found that consumers with a history of consuming insects self-reported low disgust with the intent to consume insects. La Barbera et al. [41] had also used the same instrument on Chilean consumers, which predicted their intent to consume insects as well as incorporate insects into their diets. This study was conducted to evaluate the use of the EAQ on US consumers. From the results, US consumers, from the San Luis Obispo, California area, display low disgust and food neophobia but high interest to consume insects. As a result, this suggests that the participants in this study may be potential consumers of insects and/or insect-based products in the near future.

#### 3.2.2. Beliefs, Lifestyle, Purchasing Considerations, and Product Liking

For the beliefs about consuming insects, the mean agreement scores for the statements varied between 1.96 to 4.64, for all participants, on a 5-point scale. However, the highest mean scores (above 4 mean score) were for the statements “I would consume a packaged food that contains NONVISIBLE insects”, “I would support my family members or children eating insects”, “I think that insects should be used as livestock feed”, and “I would consume livestock that was fed insects”.

This may imply that the participants would be more willing to consume insects in nonvisible forms (powders) than visible forms (whole). This further supports previous findings that some consumers may be more willing to consume nonvisible insects integrated into food products than whole insects [30,56,57,84]. This can be further supported as there was a difference in mean scores between all participants and the Hideaways for the statements “I would consume a whole roasted insect” and “…whole insect incorporated into a dish” of 0.53 and 0.49, respectively. The mean score from all participants may be influenced by the Peek-a-boos and Daredevils from the study. Rovai et al. [47] suggested that the Peek-a-boos and Daredevils groups may be more willing to adopt insect-based food products, if this included nonvisible forms, whole insects, and/or foods containing whole insects, than the Hideaways.

The mean scores regarding feeding livestock with insects further support the high mean scores for the EAQ-F statements suggesting that insects may be an alternative animal feed to supply livestock production. Indirect entomophagy, the use of insects for animal rearing, may be viable to incorporate insects into the food supply [85]. Consumers who have self-reported interest to use insects for animal meat production may suggest an intention to consume food products using indirect entomophagy in the future [31,39,41].

From the lifestyle assessment, the participants self-reported high openness to trying new foods from other cultures (4.65), high value for healthy practices including “exercising regularly”, “modifying diets”, and “looking for new foods” (mean scores 3.99, 4.22, and 4.11, respectively) and high consumption of raw fish sushi (4.26). Ruby and Rozin [82] found that high sushi intake may be an indicator of the future acceptance of insects among US and Indian consumers. This study also evaluated this factor, which may predict that US consumers, in the San Luis Obispo area, may be more willing to consume insects in the near future.

For purchasing food considerations, the participants highly rated (above 4 mean score) “cost”, “taste”, “nutritional value”, and “fresh” on a 5-point scale. “Convenience” may also be a consideration due to its high mean score (3.90). When compared to the Hideaways, the largest difference in mean scores was 0.08 between all participants and the subgroup. Pollard et al. [86], when determining factors that affect food choice of vegetable and fruit intake, suggested that food choice is a complex process that involves sensory appeal, cost, familiarity and habit, time constraints, and other factors. Placentino et al. [87] found that Italian athletes were willing to taste cricket-enriched energy bars due to the curiosity in their protein content and its textural properties. As a result, insect-based food products may need to meet these considerations to encourage their acceptance.

When evaluating products containing nonvisible insects, the participants rated the products with high liking, with “beef jerky type product” rated the lowest (3.94) and “chip, cracker, or puffed snack” rated the highest (4.28) on a 5-point scale. The mean liking scores between all participants and the Hideaways were comparable with a mean score difference of 0.11. Food appropriateness may be a consideration when incorporating insects into food products. Ardoin and Prinyawiwatkul [55] found that bakery/cereal and snack products, which include bread, bakery products such as cookies, and protein bars were deemed appropriate for the incorporation of cricket powder among US consumers. However, Lombardi et al. [34] found that pasta, a staple food, was deemed more appropriate by Italian students when compared to cookies. Meat products containing insects have also been evaluated for whether consumers are willing to consume them or if they are deemed appropriate [54,83,88,89]. Overall, food appropriateness, while not measured in this study, may be a factor when developing insect-based food products, as positive food sensory experiences with each associated product may encourage future acceptance [53,54]. From the results, the high liking across all the products evaluated by the participants may suggest their openness to incorporating nonvisible insects into these products.

### 3.3. Consumer Acceptability, Plate Waste, Insect Identification

Table 3 shows the sensory evaluation results for all participants and the Insect ID subgroup along with plate waste and the percentage of participants who selected “Yes” if the dish contained insects.

#### 3.3.1. Sausage Bruschetta Appetizer

For all participants, the Control bruschetta was rated significantly higher in liking than the CP version for aroma, overall liking, taste, flavor, and aftertaste (*p* < 0.05). No differences were found in liking for appearance and texture (*p* > 0.05). For the sausage product, there was a significant difference in liking between the Control and CP sausages for all attributes (*p* < 0.05). A higher percentage of participants indicated that the CP appetizer contained insects in the dish (*p* < 0.05). For plate waste, no differences were found when comparing the Control and CP appetizer (*p* > 0.05). Overall, the mean liking scores for the Control and CP bruschetta were from 6.80 to 7.57 and 6.50 to 7.14, respectively, across all attributes. The mean liking scores across all attributes for the Control and CP sausage were from 6.53 to 7.33 and 5.96 to 6.76, respectively.

Previous studies have found that the incorporation of insect powders in meat/sausage products reduced consumer liking. Caparros Megido et al. [83] found that beef burger patties containing mealworms (*Tenebrio molitor*) were rated lower in overall liking and other attributes by Belgian students, when compared to all-beef patties; however, the mealworm beef patties were rated higher than legume-based patties. Choi et al. [90] also found that consumer acceptance decreased as mealworm (*Tenebrio molitor*) replacement increased in pork frankfurters. For Orthoptera species, Cruz-López et al. [91] found that increasing grasshopper (*Sphenarium purpurascens*) flour decreased the overall liking of pork sausages. However, another study found that sensory characteristics did not change between different levels of mealworm replacement in pork patties except for juiciness [92].

When incorporating nonmeat ingredients into meat products such as sausages, previous studies have found that consumer acceptance may be affected [93]. Plant byproducts, such as okara, have been found to decrease sensory scores in beef patties and burgers [94,95]. However, other studies using different ingredients, such as pulses and mushroom, either maintain sensory scores or even increased them [93,96]. As a result, considerations when utilizing a nonmeat ingredient in meat products may include how it affects the overall product.

The decrease in attribute liking of the CP sausage may be attributed to the changes in the quality of the sausage product. Ho et al. [63] found that the CP sausage became darker than the Control and textural changes were found for firmness and springiness. When using cricket powder, Smarzyński et al. [97] found that the addition of cricket powder decreased the lightness of pork pâtés, and shifted the color more towards blue. Previous research using Orthoptera powders (*Acheta domesticus* and *Sphenarium purpurascens*) changed the textural properties in cased sausages [91,98]. A decrease in attributes such as taste, flavor, and texture may be the result of the cricket powder. The flavor of cricket powder has been described as woody, nutty, and even similar to pet food [89,99,100]. Not to mention, the exoskeleton of insects may lead to a gritty texture [98,99]. However, Cruz-López et al. [91] and Keto et al. [101] suggest that the negative effects on these attributes may be offset with the addition of spices and flavorings that may improve the acceptance of meat products containing insects. From the results, the sausage and bruschetta were acceptable for the participants (6.50 and 7.14, respectively, in overall liking). In addition, the sausage product was served as a component in the bruschetta appetizer and may have had an effect as no differences were found in liking scores for appearance and texture (*p* > 0.05). Therefore, when using insect powders in meat products, the negative changes in sensory characteristics may be mitigated through formulation and product delivery methods.

#### 3.3.2. Pasta Entrée

The CP pasta entrée rated higher (*p <* 0.05) in overall liking than the Control by all participants. No differences were found for the other hedonic attributes, “Yes, it contained insects” percentage, and plate waste (*p* > 0.05). Overall, the mean liking scores for the Control and CP pasta were from 6.34 to 7.01 and 6.44 to 7.05, respectively, across all attributes.

Previous studies have evaluated consumer liking of pasta containing insect ingredients [102,103,104,105]. Duda et al. [102] found that 5% usage of cricket powder in pasta was met with high acceptance by consumers. Results from this study may also support this as the participants scored the CP pasta, which contains 5% cricket powder (Table A2), higher in overall liking than the Control (*p* < 0.05). However, results from Duda et al. [102] found that insect powders may decrease acceptance as insect powder usage increases. Çabuk et al. [104] and Jakab et al. [105] support this, as the sensory characteristics were reduced with the addition of insect powders in egg pasta and millet pasta, respectively. Though sensory liking may decrease, the use of insect powders in pasta, especially in healthier alternatives, may provide new market opportunities. Biro et al. [103] found that Hungarian students preferred buckwheat pasta containing silkworm (*Bombyx mori*) powder more than the control buckwheat pasta.

To further support this, previous research has found that insect-based pasta has been found to resemble whole wheat pasta which is considered a healthier option to conventional pasta [102,106]. This may be a result of the cricket powder shifting the color of pasta to be dark in appearance [102,106]. To mitigate this, Ho et al. [63] developed the Control and CP pasta formulations with caramel coloring to limit noticeable differences in appearance that the participants may detect. Lombardi et al. [34] mentioned that pasta is considered a staple food with a low hedonic value. However, this can be mitigated through pasta preparation such as the addition of dressings and sauces [34,83,107]. From the results, both the Control and CP pasta were rated as acceptable by the participants. Therefore, the use of insects may provide opportunities to develop health-oriented pasta products.

#### 3.3.3. Brownie Dessert

For all participants, the CP brownie was rated significantly higher in liking for aroma than the Control (*p* < 0.05). However, the CP dessert was rated significantly lower in taste, texture, and aftertaste than the Control (*p* < 0.05). No differences were found for the other attributes (appearance and flavor), “Yes, it contained insects” percentage, and plate waste (*p* > 0.05). Overall, the mean liking scores for the Control and CP brownies were from 7.33 to 7.87 and 6.89 to 7.67, respectively, across all attributes evaluated.

The use of cricket powder in brownies and other sweet bakery products may be a viable application. As mentioned, cricket powder has been found to have a nutty and woody flavor [99,100]. From a product development perspective, using cricket powder in chocolate-based products, such as brownies, may be a method to utilize the sensory characteristics of cricket and other insect powders. Castro Delgado et al. [108] found that cricket powder in chocolate chip cookies had no effect on liking when compared to their control among US consumers. The use of chocolate may mitigate the negative effects of insect powders on bakery products, as it may reduce the shift in color and possibly lead to a decrease in liking in appearance/color [109,110].

Gurdian et al. [52] evaluated the cricket powder replacement in brownies, and the brownies containing cricket powder were rated lower in liking than the brownies that did not. However, Gurdian et al. [52] suggested that this may be due to the effect of the formulation of the brownies. From the results, although there were differences in attribute liking, the CP brownie may be as acceptable as the Control; this may be due to the formulation as well as the product delivery method. Due to the gritty texture of cricket powder [98,99], Ho et al. [63] further ground the cricket powder used in the formulation. Cricket powder has been found to change the textural properties of bakery products which may be due to the prevention of gluten formation and/or changes in amylose/amylopectin proportions [109,110,111]. As a result, methods to mitigate the effects of insect powders on bakery products may be a consideration, including formulation.

#### 3.3.4. Insect ID Subgroup

For the bruschetta and sausage, the Insect ID subgroup (*n* = 48) rated the Control higher in liking than the CP version for all attributes tested (*p* < 0.05). There was no difference for plate waste (*p* > 0.05). For the pasta, no differences in attribute liking were found (*p* > 0.05), but there was a higher amount of plate waste (*p* < 0.05) for the CP pasta than the Control by the subgroup (*n* = 32). The Control brownie was rated higher in liking (*p* < 0.05) for overall liking, taste, texture, flavor, and aftertaste by the Insect ID subgroup (*n* = 43). No differences in liking were found for appearance and aroma (*p* > 0.05). There was also higher plate waste (*p* < 0.05) collected from the CP brownie than the Control by the subgroup.

There were more differences in attributes evaluated between the bruschetta appetizer and brownies within the Insect ID subgroup (*p* < 0.05). Not to mention, the differences in mean liking scores of the Control and CP bruschetta, sausage, and brownies for the Insect ID subgroup were comparatively higher than by all participants. The largest difference in mean liking scores for the bruschetta, when evaluated by all participants and the Insect ID subgroup, was 0.47 to 0.75, respectively, for taste. For the sausage, the largest difference was 0.84 (all participants) to 1.23 (Insect ID subgroup) for texture. For the brownie, the largest difference between all participants and the Insect ID subgroup was 0.25 to 0.85, respectively, for flavor. However, there was no difference found in liking for flavor when evaluated by all participants (*p* > 0.05).

This study did not inform the participants which products/dishes did or did not contain insects. Previous research has found that informing consumers that products that contained insects may lead to lower expected liking scores prior to tasting [51,52,54,89]; however, expected liking may not necessarily affect future acceptance. Schouteten et al. [89] found that informed conditions did not affect the sensory profile between beef burgers and insect-based burgers when evaluated, and the low acceptance of the insect-based burger was due to low sensory quality. Although there were more differences in attribute liking for the Insect ID subgroups, the subsamples may be too small (*n* = 48 for the bruschetta and sausage; *n* = 43 for the brownie). Not to mention, the Insect ID subgroup that selected “Yes, it contained insects” may have selected the correct answer by chance. For the purposes of this study, the authors wanted to determine whether the participants were able to detect if the products contained insects. From the results, most of the participants were unaware that the products/dishes evaluated had even contained insects. At the end of the study, some of the participants commented that they did not believe any of the products tested even contained any insects.

### 3.4. Emotional Response

#### 3.4.1. Emotional Response before and after Evaluation

**Figure 2:**
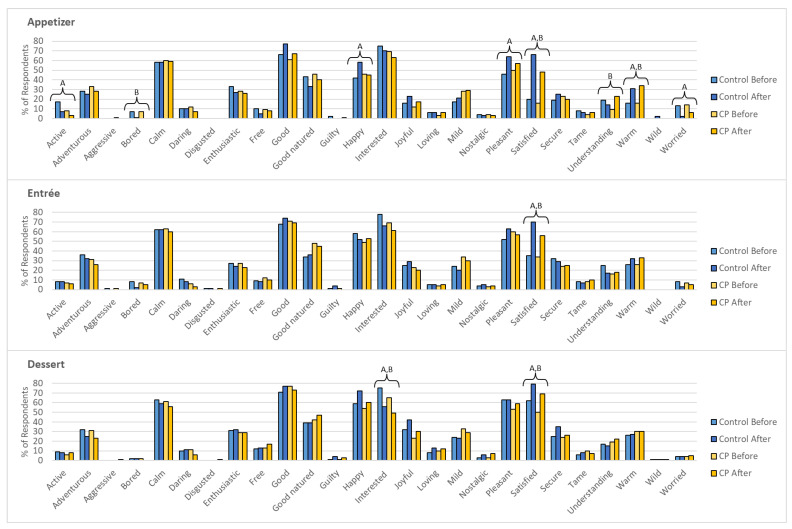
The percentage of participants who selected an emotion term before and after each dish evaluation. Bar graphs were colored blue for the Control dishes (*n* = 106) and yellow for the CP dishes (*n* = 103). Emotions that were denoted with a number were found to have a significant difference between the percentages of participants who selected the emotion term before and after consumption, using a two-tailed *z*-test *(p* < 0.05), for (A) the Control dish and (B) the CP dish.

Figure 2 shows the emotion term selection before and after evaluating each dish. For the Control appetizer, there was a significant increase (*p* < 0.05) in the selection of positive emotions by participants for happy, pleasant, satisfied, and warm after consumption. There was a significant decrease (*p* < 0.05) in emotion term selection by participants for active (positive) and worried (negative) after consumption. For the CP appetizer, there was an increase (*p* < 0.05) in the selection of the satisfied and warm (positive) as well as understanding (neutral) after consumption. There was a significant decrease (*p* < 0.05) in the selection for bored (neutral) for the CP (from 7% to 0%); however, there was also a decrease in the selection of bored for the Control appetizer (from 7% to 1%), though no significance was found (*p* > 0.05).

For the entrée, there was a significant increase (*p* < 0.05) in the selection of the term satisfied (positive) after consumption for both the Control and CP versions. No change in selection was found for the other emotion terms (*p* > 0.05).

When evaluating the dessert, there was an increase (*p* < 0.05) in selection for the positive terms interested and satisfied. No change in selection was found for the other emotion terms for the dessert (*p* > 0.05).

Measuring emotional responses elicited by food may expand findings during sensory evaluation to better predict the future acceptance of food products [112,113,114,115]. The use of emotion terms/lexicons has suggested that the selection of positive (happy, active, interested, satisfied, warm, etc.) and neutral/unclassified (understanding, daring, wild) emotion terms were associated with highly liked products, while lowly liked products were associated with negative emotions (disgusted, bored, and worried) [74,115].

From the results, the participant selected positive emotions such as calm, good, happy, interested, pleasant, satisfied, and warm. The products/dishes may have elicited more positive emotions than negative emotions after consumption of each dish. This can be supported as the selection of satisfied increased (*p* < 0.05) after the consumption/evaluation of all dishes. This emotion may relate to the food satisfaction participants may perceive in certain eating situations, which may relate to food appropriateness and acceptance [64]. To the authors’ knowledge, the relation between emotions such as satisfied and food satisfaction has not been studied, something which may give more insight in the future acceptance of new food products such as insects. However, the selection of the term interested decreased after the evaluation of all the dishes, which was significant for the dessert (*p* < 0.05) but not for the appetizer and entrée (*p* > 0.05). Overall, positive emotions were elicited throughout the study which may support the high liking scores of the products/dishes.

Regarding insects as food, it has been found that insects elicit negative emotions such as disgust when people are aware that the product contains insects [84]. From this study, the products/dishes evaluated showed limited negative emotions throughout the courses served in this study. There was little to no selection of negative emotion terms, including disgusted and bored by participants when evaluating all products/dishes. At most, 1% of participants self-reported the term disgusted for the Control and CP entrée and CP dessert. Schouteten et al. [89] found that when participants were informed that the insect-based burgers contained insects, negative emotions, such as disgust, were reported. Le Goff and Delarue [71] found that participants expressed more negative facial expressions when they were informed that the chip products were “enriched with cricket protein” than when they were not. This may be due to information regarding food products that may elicit negative emotions associated with that product, such as insects [84,113,114]. However, negative emotions consumers may experience have been found to be reduced once they taste the product [71,89]. This can be further supported as there was a decrease in the selection of the term worried (*p* < 0.05) after the consumption of the Control appetizer. Emotion terms, such as worried, have been found to be associated with novel foods due to concerns of safety and feelings of distrust [70]. The participants may have experienced worry when evaluating the Control appetizer as this was the first product/dish being evaluated in the overall study with the participants unaware of which products may contain insects.

#### 3.4.2. Changes in Emotional Response

**Figure 3:**
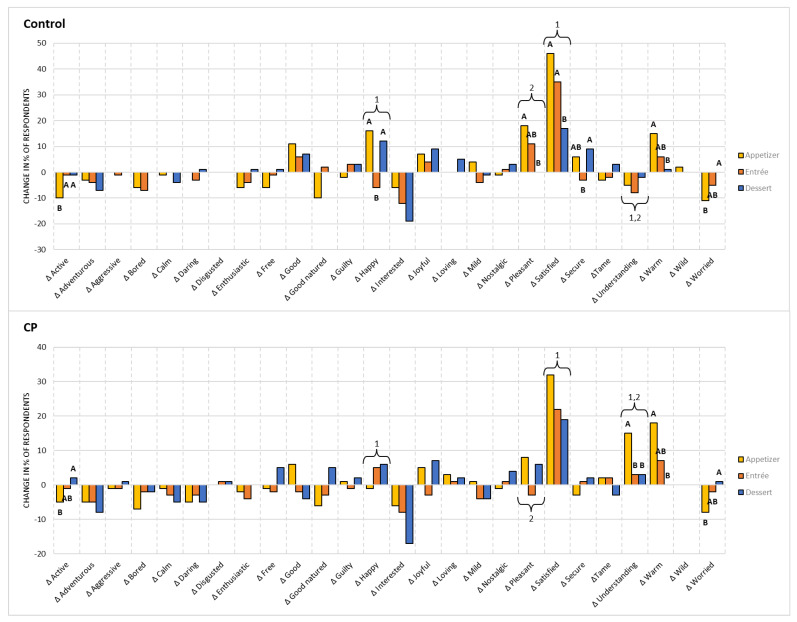
ΔEmotion for each emotion term before and after evaluating a dish for both the Control and CP versions. Emotions terms denoted by a number (shown on both versions) were found to have a significant difference (*p* < 0.05) in the change in emotion term selection between the Control and CP, with (1) being the Appetizer and (2) the Entrée, using a two-way ANOVA blocked on Treatment (Control and CP) and Participant. No differences were found for the change in emotion selection between the Control and CP (*p* > 0.05). Control and/or CP dishes with differentiating letters, for each emotion term, were found to be significantly different (*p* < 0.05) using a two-way ANOVA, blocked on Product (sausage, pasta, and brownie) and Participant, followed by Tukey’s means comparisons test.

To better understand the emotions the participants self-reported, Figure 3 shows the change in emotion term selection throughout the three-course meal for each week (Control and CP). For the Control dishes, there was a significant difference between the changes in emotion selection for active, happy, pleasant, pleasant, satisfied, secure, understanding, warm, and worried (*p* < 0.05). With the CP dishes, changes in emotion selection for the terms active, understanding, warm, and worried were significantly different between the dishes (*p* < 0.05). For the appetizer, the change in the selection of happy was positive for the Control, while it was negative for the CP version (*p <* 0.05). The change in selection of satisfied was greater for the Control appetizer than the CP appetizer (*p <* 0.05). For the entrée, the change in the selection of pleasant was positive for the Control, while it was negative for the CP entrée (*p <* 0.05). For both the appetizer and entrée, the change in selection for understanding was more positive for both CP versions, while it was negative for both Controls.

ΔEmotion was analyzed to understand and visualize emotional responses before and after the consumption of each dish. The selection of positive emotions increased for good, happy, pleasant, satisfied, secure, and warm for the Control and CP dishes. However, the change in the selection of positive emotions was less prominent, with a decrease in the selection of emotions terms, for the CP when compared to Control. This can be supported due to a difference between the changes in the selection of happy and satisfied (*p* < 0.05). King et al. [116] suggested that the number of samples tested may affect the intensity of certain emotion terms including interested and satisfied. Not to mention, when evaluating facial expressions, de Wijk et al. [117] found that repeated exposure when evaluating the same products may affect emotional responses over time. Limitations of using emotional response measurements, such as EsSense Profile^®^ or other emotion term lists, may include heavy cognitive involvement by the participants, which may lead to fatigue during product evaluation. Although there was no difference (*p* > 0.05), there was a negative change in the selection of interested throughout the three-course meal for both the Control and CP versions. The feeling of interest may be associated with the curiosity the consumers experienced once the dish was served to them. Once the participants had consumed and evaluated the product, the loss of interest in the dish may be due to that associated curiosity being fulfilled.

For the CP dishes, there was an increase in the selection of understanding while there was a decrease for the Control dishes. This occurrence was the highest for the CP appetizer (*p* < 0.05), which may support that the participants were able to detect that the dish did contain insects. However, the increase in understanding, an unclassified/neutral emotion, may be an indicator that the CP dishes may have not elicited negative emotions. This can be further supported, as there was a decrease in the selection of worried by the participants.

The emotion of satisfied may be of interest as the selection of this term was the most prominent among all the terms selected. In a food service setting, previous studies have found that feelings of satisfaction and contentment when consuming foods may be factors for food choice and consumption [65,118]. When evaluating food products through full-size servings, fulfilment-related emotions, such as satisfaction, may need to be further studied to understand the future acceptance of food products. From the results, there was a decrease in the change of satisfied over the three-course meals (*p* < 0.05). This may relate to the changes in perceived hunger/satiety, which is later explained, that consumers may experience when consuming multiple dishes in a normal eating situation. As a result, further research may be necessary to understand emotional responses during eating situations, such as a three-course meal.

Overall, throughout the three-course meal, the participants selected more positive emotion terms when compared to the negative terms provided for both the Control and CP dishes. Previous research supports that reducing negative emotions associated with insect-based food products but increasing positive emotions may increase future consumption of insects [27,50,84]. The frequency of positive emotions when evaluating the products may support that the CP products were as acceptable as the Control products.

### 3.5. Relationship between Consumer Acceptability, Plate Waste, and Satiety

#### 3.5.1. Satiety

**Figure 4:**
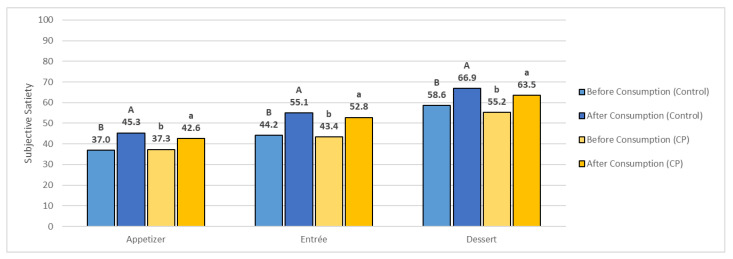
Mean subjective satiety reported by the participants, using the SLIM scale [76], before and after the consumption of each dish. Bar graphs were colored blue for the Control dishes and yellow for the CP dishes. Differentiating letters (uppercase for Control, lowercase for CP) shows a significant difference was found between the before and after subjective satiety using a two-way ANOVA, blocked on Product Evaluation (Product Before and After) and Participant, for each dish (*p* < 0.05).

Figure 4 shows the subjective satiety reported by the participants for each week during product/dish testing during a three-course meal. Overall, there was a significant difference in reported mean satiety before and after the consumption/evaluation of each dish for both the Control and CP (*p* < 0.05). From the results, the participants’ perceived satiety was increasing, suggesting that the participants’ perceived fullness was increasing, or perceived hunger was decreasing, as they continued to consume each dish.

Satiety, or perceived/subjective satiety, has been studied as a driver of liking and food intake for food product development [119,120]. Satiety may be affected by the sensory properties of the product, including texture, as well as the serving size, and whether the product is large-sized or bite-size [121,122]. The change in perceived satiety after consumption has been found to be a predictor of purchase intent and acceptance of a product [119]. To the authors’ knowledge, there is limited research on measuring the acceptability of products, especially in an eating situation where participants are served in a three-course meal. Overall, subjective/perceived satiety increased over the course of product/dish evaluation, which is to be expected as the participants were allowed to eat as much as they liked.

#### 3.5.2. Consumer Liking, Satiety, and Plate Waste

Table 4 shows the correlation coefficients between the attribute liking scores (including all products/dishes), satiety variables, and plate waste. For the Control, there were significant negative relationships between flavor liking and ΔSatiety, as well as attribute liking scores (overall liking, taste, and texture) and plate waste (*p* < 0.05). Although no significance was found (*p >* 0.05), attribute liking scores (aroma, flavor, aftertaste) were found to have a high negative correlation with ΔSatiety and plate waste (−1 > *r* > −0.70). There were significant positive correlations between appearance liking and satiety before and after *(p* < 0.05). A moderate positive correlation was found between texture liking and satiety before (0.50 > *r* > 0.70), though no significance (*p* > 0.05).

For the CP dishes, no significant relationships were found (*p* > 0.05). However, appearance liking had a high positive correlation with Δsatiety (0.70 > *r* > 1), while aftertaste liking was found to moderate positive correlation with ΔSatiety (0.50 > *r* > 0.70). There was a high positive correlation between attribute liking (appearance, aroma, overall liking, taste, texture, and aftertaste) and subjective satiety for both before and after (0.70 > *r* > 1). There was also a moderate negative correlation between taste liking and plate waste (−0.70 > *r* > −0.50).

When Control and CP are combined, a significant positive correlation was found between appearance liking and satiety before and after (*p* < 0.05). A moderate positive correlation was found between appearance liking and ΔSatiety, as well as for attribute liking (aroma, overall liking, texture, and aftertaste) and satiety before and after consumption (0.50 > *r* > 0.70). There was a significant negative relationship between taste liking and plate waste *(p* < 0.05). However, no significance *(p* > 0.05) attribute liking for aroma, overall liking, texture, flavor, and aftertaste, were found to have a moderate negative correlation with plate waste (−0.70 > *r* > −0.50).

Plate waste has been used to assess the implementation of new food products in food service settings, especially for child nutrition programs [123,124,125,126]. Plate waste has also been determined to be a predictor of food choice regarding portion control [127,128]. From the results, a negative relationship between consumer liking scores and plate waste was found. As plate waste increased, the liking scores decreased across the products/dishes evaluated. When introducing novel foods in US school lunch programs, it was found that plant-based meals increased plate waste by students [129]. However, this may be due to the unfamiliarity of these foods. Martins et al. [130] suggested that the sensory characteristics that increase satisfaction with certain meals may influence the food waste produced by US students as well.

Regarding subjective satiety, the results may suggest that perceived satiety may be a factor in consumer acceptance. The perceived satiety consumers may experience when evaluating a product possibly affects their evaluation. As ΔSatiety increased, consumer liking of the attributes decreased, while satiety before and after positively affected consumer liking. However, Yeomans et al. [131] suggested that flavor and palatability of porridges may be a better predictor of the intent to purchase of a product than perceived satiety. Further research into perceived satiety over the course of product evaluation may be necessary.

Although this may be a novel approach, plate waste may give insight into the future consumer acceptance of new food products. Typically, product evaluation involved providing participants with small portions of the product to be tested. For the purposes of this study, the products were served in a manner that consumers may typically consume in certain eating situations, such as a three-course meal. From the results, plate waste may be an indicator of future acceptance as there was a moderate negative correlation between both the Control and CP dishes (combined) and plate waste.

## 4. Conclusions

This study was designed to evaluate potential products containing insect powders. As a result, this was a blind study where participants were not informed which products contained insects. The products were measured for consumer acceptability as well as measuring emotional response, satiety, and plate waste as factors in the future acceptance of these products. The US consumers recruited for this study were found to be potential early adopters of insect-based foods and who may be open to different food products, especially for the products tested, containing nonvisible insects. During consumer acceptability testing, though there was a difference in attribute liking, the CP products/dishes were still considered acceptable by the participants when compared to the Controls. These findings provide more insight into the products developed by Ho et al. [63], and that formulation and serving methods may increase the overall acceptability of food products containing insect powders. This can also be further supported by the fact that the emotional responses evaluated as more positive emotion terms, such as satisfied and interested, were selected more than negative emotions, such as disgusted and worried. Plate waste may also assess the performance of these in certain eating situations. Consumer liking may relate to how much plate waste is produced by consumers and predict acceptance of novel foods such as insect-based foods. The findings from this study may provide a novel approach to assessing new food products by measuring different factors during the product consumption experience.

## Figures and Tables

**Table 1:** Sociodemographics, insect familiarity, and cluster assignments of the participants (*n* = 106) who responded to the demographics questionnaire. Proportions are rounded to the nearest tenth place.

	Proportions (%)	Base Size *n*
**Total**	-	106
**Gender Group**		
Male	31.1	33
Female	67.9	72
Prefer not to say	0.9	1
**Age Group**		
18–24	16.0	17
25–34	18.9	20
35–44	28.3	30
45–54	17.9	19
55–64	17.0	18
65 and Over	1.9	2
**Ethnicity**		
White or Caucasian	70.8	75
Hispanic or Latino	11.3	12
Black or African American	0	0
Native American or American Indian	0.9	1
Asian or Pacific Islander	15.1	16
Other	1.9	2
**Highest Education Level**		
Some High School	0	0
High School Graduate or Equivalent	0	0
Some College	16.0	17
Trade, Technical or Vocational Training	0.9	1
Associate Degree	5.7	6
Bachelor’s Degree	40.6	43
Master’s Degree	27.4	29
Professional Degree	0	0
Doctorate Degree	9.4	10
**Diet**		
No restrictions	91.5	97
Limited animal products	7.5	8
Vegetarian	0.9	1
Vegan	0	0
Other	0	0
**Insect Familiarity Statements**		
Yes, I have heard of the eating of insects and I know what it means	84.0	89
I have heard of the eating of insects but actually don’t know what it means	15.1	16
No, I have never heard of the eating of insects	0.9	1
**Cluster Group Assignments**		
No-Thank-Yous	6.6	7
Hideaways	57.5	61
Daredevils	10.4	11
Peek-a-boos	11.3	12
Flat	14.2	15

**Table 2:** Mean scores for the EAQ factors, FNS, belief about consuming insects, purchasing considerations, and liking of products containing nonvisible insects for all participants and the Hideaways subgroup.

Statements/Items	Mean Score	
EAQ—Disgust (EAQ-D) ^7A^	All (*n* = 106)	Hideaways (*n* = 61)
I would be disgusted to eat any dish with insects	2.26	2.38
Thinking about the flavor that a bug might have sickens me	2.79	3.03
If I ate a dish and then came to know that there were insects among the ingredients, I would be disgusted	2.11	2.23
I would avoid eating a dish with insects among the ingredients: even if it was cooked by a famous chef	1.80	1.84
I would be bothered by finding dishes cooked with insects on a restaurant menu	2.03	2.15
**EAQ—Interest (EAQ-I) ^7A^**		
I’d be curious to taste a dish with insects, if cooked well	6.13	6.07
In special circumstances: I might try to eat a dish of insects	5.75	5.59
At a dinner with friends I would try new foods prepared with insect flour	6.14	6.15
**EAQ—Animal Feed (EAQ-F) ^7A^**		
Using insects as feed is a good way of producing meat	5.47	5.36
I think it is fine to give insect-based to fish that are farmed for human consumption	6.14	6.25
**Food Neophobia ^7A^**		
At dinner parties, I will try a new food (R)	1.25	1.25
Ethnic food looks too weird to eat	1.65	1.61
I am afraid to eat things I have never had before	2.14	2.18
I am constantly sampling new and different foods (R)	2.24	2.44
I am very particular about the foods I will eat (R)	3.03	3.21
I do not trust new foods	2.18	2.28
I like foods from different countries (R)	1.58	1.70
I like to try new ethnic restaurants (R)	1.73	1.82
I will eat almost anything (R)	2.29	2.56
If I do not know what is in a food, I won’t try it	2.73	2.84
Beliefs about Consuming Insects ^5A,^*		
I would consume a live insect	1.96	1.66
I would consume a whole roasted insect	2.99	2.46
I would consume a whole insect incorporated into a dish	3.38	2.89
I would consume a packaged food that contains WHOLE insects	2.81	2.28
I would consume a packaged food that contains NONVISIBLE insects	4.42	4.38
I would support my family members or children eating insects	4.42	4.39
I think more people should consume insects	3.88	3.79
I think that insects should be used as livestock feed	4.28	4.26
I would consume livestock that was fed insects	4.64	4.64
Lifestyle Assessment ^5A,^*		
I exercise regularly	3.99	4.02
I enjoy eating foods and ingredients from other cultures	4.65	4.64
I and or other members of my household have food allergies that prevent us from eating certain foods	1.72	1.80
I am willing to pay more for all natural or organic foods	3.52	3.56
I am concerned about the environmental impact of the foods I eat	3.82	3.74
I mostly eat a plant-based diet	2.32	2.28
I am willing to modify my diet in order to be healthier	4.22	4.26
I am always looking for new foods to help me live a healthier life	4.11	4.07
It is difficult for me to understand what products are truly nutritious and or healthy for me and my family	2.43	2.48
I am loyal to brands that I like	3.67	3.66
I eat raw fish sushi	4.26	4.10
Purchasing of Food Considerations ^5A,^*		
Cost	4.38	4.39
Dietary restrictions	2.24	2.30
Convenience	3.90	3.85
Sustainable ingredients	3.54	3.49
Organic	3.11	3.15
Non-GMO	2.95	2.95
Taste	4.75	4.80
Nutritional value	4.15	4.16
Sustainably produced	3.47	3.52
Local	3.73	3.70
Fresh	4.29	4.31
Minimally processed	3.68	3.72
Natural	3.62	3.54
Preservative free	3.37	3.36
Brand reputation	3.38	3.33
Products Containing Nonvisible Insects ^5L,^*		
Chip, cracker, or puffed snack	4.28	4.28
Pasta product	4.24	4.30
Dessert product, cookie or cake	4.08	4.00
Beef jerky type product	3.94	3.87
Protein shake or powder	4.18	4.20
Cereal granola	4.07	3.98
Baked product, bread	4.16	4.05
Meat analog, sausage or ground meat	4.20	4.15
Protein bar	4.25	4.28

^7A^ Participants were asked to rate on a 7-point agreement scale (1 = Completely Disagree; 7 = Completely Agree). ^5A^ Participants were asked to rate on a 5-point agreement scale (1 = Completely Disagree; 5 = Completely Agree). ^5L^ Participants were asked to rate on a 5-point Likert scale (1 = Dislike very much; 5 = Like very much). (R) Food neophilia statement scores from the FNS were reversed. * The set of statements were developed by Rovai et al. [47].

**Table 3:** Mean attribute liking scores, percentage of participants who selected “Yes” the dish contained insects, and plate waste for the dishes/products evaluated (bruschetta, sausage, pasta, and brownies) for all participants and the Insect ID subgroup. Base sizes (*n*) of participants that evaluated each product/dish are also shown. Parameters colored (blue for ALL participants, yellow for the Insect ID subgroup) and denoted were found to be significantly different (*p* < 0.05) using a two-way ANOVA, blocked on Treatment and Participants, for hedonic liking and plate waste. A two-tailed *z*-test was used for the “Yes, it contained insects” percentage.

Product and Attributes	All Participants		Insect ID Subgroup	
	Control	CP	Control	CP
**Bruschetta**	*n* = 106	*n* = 103	*n* = 48	
Appearance ^2^	6.80	6.67	6.83	6.40
Aroma ^1,2^	7.42	7.13	7.52	6.98
Overall ^1,2^	7.57	7.14	7.65	6.96
Taste ^1,2^	7.56	7.09	7.58	6.83
Texture ^2^	7.20	6.97	7.31	6.62
Flavor ^1,2^	7.49	7.08	7.56	6.87
Aftertaste ^1,2^	7.11	6.50	6.98	6.29
Contained Insects? Yes (%) ^1^	22	47	-	-
Plate Waste (%)	31.0	32.7	31.1	33.4
**Sausage**			*n* = 48	
Appearance ^1,2^	6.53	5.96	6.67	5.90
Aroma ^1,2^	7.20	6.76	7.33	6.69
Overall ^1,2^	7.33	6.50	7.42	6.21
Taste ^1,2^	7.31	6.71	7.42	6.40
Texture ^1,2^	7.18	6.34	7.23	6.00
Flavor ^1,2^	7.29	6.56	7.19	6.23
Aftertaste ^1,2^	6.80	6.11	6.75	5.83
**Pasta**			*n* = 32	
Appearance	6.97	6.98	6.88	6.72
Aroma	7.01	7.05	6.69	6.56
Overall ^1^	6.60	6.90	6.34	6.50
Taste	6.55	6.74	6.34	6.34
Texture	6.67	6.70	6.41	6.25
Flavor	6.69	6.82	6.69	6.56
Aftertaste	6.34	6.44	6.32	6.22
Contained Insects? Yes (%)	29	31	-	-
Plate Waste (%) ^2^	40.9	44.2	32	39.4
**Brownie**			*n* = 43	
Appearance	7.45	7.42	7.54	7.44
Aroma ^1^	7.42	7.67	7.69	7.81
Overall ^2^	7.87	7.65	7.96	7.30
Taste ^1,2^	7.78	7.41	7.92	7.14
Texture ^1,2^	7.33	6.98	7.43	6.77
Flavor ^2^	7.75	7.50	7.90	7.05
Aftertaste ^1,2^	7.36	6.89	7.34	6.58
Contained Insects? Yes (%)	29	42	-	-
Plate Waste (%) ^2^	27.5	30.7	23.1	30.7

^1^ A significant difference was found between the Control and CP versions of that product by ALL participants from the study (*p* < 0.05). ^2^ A significant difference was found between the Control and CP versions of that product by participants from the Insect ID subgroup per dish/product (*p* < 0.05).

**Table 4:** Correlation coefficients between attribute liking scores, ΔSatiety, satiety before and after, and plate waste. Coefficients were either colored blue if *r* > 0.50 or yellow if *r* < −0.50. Correlation coefficients denoted were found to be significant (*p* < 0.05).

**Control**				
**Attributes**	**Δsatiety**	**Satiety before**	**Satiety after**	**Plate Waste (%)**
Appearance	0.06	0.96 *	0.96 *	−0.22
Aroma	−0.85	0.26	0.15	−0.89
Overall	−0.91	0.37	0.25	−0.98 *
Taste	−0.92	0.32	0.20	−0.97 *
Texture	−0.83	0.55	0.44	−0.96 *
Flavor	−0.97 *	−0.08	−0.20	−0.91
Aftertaste	−0.85	0.43	0.32	−0.94
**CP**				
**Attributes**	**Δsatiety**	**Satiety before**	**Satiety after**	**Plate Waste (%)**
Appearance	0.75	0.85	0.88	0.11
Aroma	0.47	0.90	0.86	−0.32
Overall	0.40	0.81	0.77	−0.33
Taste	0.25	0.80	0.73	−0.52
Texture	0.40	0.79	0.76	−0.31
Flavor	−0.05	0.31	0.25	−0.40
Aftertaste	0.51	0.86	0.84	−0.22
**Control and CP Combined**				
**Attributes**	**Δsatiety**	**Satiety before**	**Satiety after**	**Plate Waste (%)**
Appearance	0.57	0.84 *	0.88 *	−0.06
Aroma	0.23	0.60	0.59	−0.51
Overall	0.06	0.54	0.51	−0.68
Taste	0.00	0.49	0.45	−0.77 *
Texture	0.11	0.63	0.60	−0.66
Flavor	−0.02	0.08	0.07	−0.68
Aftertaste	0.20	0.53	0.53	−0.63

* Correlation coefficients were found to be significant from the table of critical values for Pearson’s *r* correlations (*p <* 0.05).

## Data Availability

The data presented in this study are available on request from the corresponding author. The data are not publicly available due to privacy and ethical restrictions.

## Appendix A

Appetizer—Sausage bruschetta with an onion and pepper topping

**Table A1:** Ingredients used and their sources for the Sausage with Peppers and Onions Bruschetta. The sausage component shows the formulations for both the Control and CP versions. Usage levels (%) shown are the percentages used for each dish component. Percentages were rounded to the nearest hundredths.

Dish Component	Ingredient	Usage Level (%)		Source
**Sausage**		**Control**	**CP**	
	Pork Shoulder	62.29	56.06	Cal Poly Meats, San Luis Obispo, CA, USA
	Pork Backfat	31.15	31.15	Cal Poly Meats, San Luis Obispo, CA, USA
	Cricket Powder	-	6.23	JR Unique Foods, Udon Thani, Thailand
	Fennel Seed, Ground	0.91	0.91	Private Selection, Kroger Co., Cincinnati, OH, USA
	Dried Basil	0.13	0.13	First Street, Amerifoods Trading Co., Los Angeles, CA, USA
	Dried Oregano	0.16	0.16	First Street, Amerifoods Trading Co., Los Angeles, CA, USA
	Crushed Red Chili Pepper	0.17	0.17	Kirkland Signature, Costco Wholesale Co., Seattle, WA, USA
	Garlic, Minced	3.74	3.74	Christopher Ranch, Gilroy, CA, USA
	Onion Powder	0.37	0.37	First Street, Amerifoods Trading Co., Los Angeles, CA, USA
	Black Pepper, Ground	0.09	0.09	First Street, Amerifoods Trading Co., Los Angeles, CA, USA
	Salt	0.76	0.76	First Street, Amerifoods Trading Co., Los Angeles, CA, USA
	Dried Thyme	0.14	0.14	First Street, Amerifoods Trading Co., Los Angeles, CA, USA
	Dried Rosemary	0.10	0.10	Private Selection, Kroger Co., Cincinnati, OH, USA
**Bruschetta (Toasted Bread)**	French Bread, Sliced	-		First Street, Amerifoods Trading Co., Los Angeles, CA, USA
	Grated Parmesan Cheese	-		First Street, Amerifoods Trading Co., Los Angeles, CA, USA
**Onion and Pepper Topping**	Yellow Onions, Sliced	54.50		Western Onion, Camarillo, CA, USA
	Extra Virgin Olive Oil	4.07		Kirkland Signature, Costco Wholesale Co., Seattle, WA, USA
	Canned Roasted Red Peppers, Sliced	41.42		First Street, Amerifoods Trading Co., Los Angeles, CA, USA

Entrée—Pasta with a charred tomato sauce

**Table A2:** Ingredients used and their sources for the pasta entrée. The pasta component shows the formulations for both the Control and CP versions. Usage levels (%) shown are the percentages used for each dish component. Percentages were rounded to the nearest hundredths.

Dish Component	Ingredient	Use Level (%)		Source
**Pasta**		**Control**	**CP**	
	Durum Wheat Semolina	61.41	73.87	Miller Milling Company, Fresno, CA, USA
	Whole Wheat Flour	14.41	-	King Arthur Flour, Norwich, VT, USA
	Cricket Powder	-	5.00	JR Unique Foods, Udon Thani, Thailand
	Powdered Caramel Coloring (P600)	0.10	0.075	Sethness Products Company, Clinton, IA, USA
	Water	24.08	21.05	-
**Tomato Sauce**	Canned Tomatoes, Diced	76.37		First Street, Amerifoods Trading Co., Los Angeles, CA, USA
	Extra Virgin Olive Oil, 1st Addition	5.81		Kirkland Signature, Costco Wholesale Co., Seattle, WA, USA
	Fresh Garlic, Minced	7.69		Christopher Ranch, Gilroy, CA, USA
	Salt	0.70		First Street, Amerifoods Trading Co., Los Angeles, CA, USA
	Black Pepper, Ground	0.17		First Street, Amerifoods Trading Co., Los Angeles, CA, USA
	Extra Virgin Olive Oil, 2nd Addition	9.27		Kirkland Signature, Costco Wholesale Co., Seattle, WA, USA
**Topping**	Grated Parmesan Cheese	-		First Street, Amerifoods Trading Co., Los Angeles, CA, USA
	Fresh Basil, Chiffonade	-		Simple Truth Organic, Kroger Co., Cincinnati, OH, USA

Dessert—Brownie trifle dessert with a berry sauce

**Table A3:** Ingredients used and their sources for the brownie dessert. The pasta component shows the formulations for both the Control and CP versions. Usage levels (%) shown are the percentages used for each dish component. Percentages were rounded to the nearest hundredths.

Dish Component	Ingredient	Usage Level (%)		Source
**Brownie**		**Control**	**CP**	
	Dutch Cocoa Powder	4.91	4.91	The Hershey Co., Hershey, PA, USA
	Bittersweet Chocolate	11.86	11.86	Puratos Chocolate USA, Kenosha, WI, USA
	Unsalted Butter	26.18	26.18	Kirkland Signature, Costco Wholesale Co., Seattle, WA, USA
	Eggs, Beaten	16.36	16.36	The Kroger Co., Cincinnati, OH, USA
	Granulated Sugar	21.27	21.27	Sysco Corp., Houston, TX, USA
	Salt	0.36	0.36	First Street, Smart & Final, Commerce, CA, USA
	Vanilla Extract	0.57	0.57	Cook Flavoring Co., Paso Robles, CA, USA
	Bread Flour	9.41	2.41	General Mills Operations Inc., Minneapolis, MN, USA
	Cricket Powder	-	7.00	JR Unique Foods, Udon Thani, Thailand
	Bittersweet Chocolate Chunks	9.07	9.07	Puratos Chocolate USA, Kenosha, WI, USA
**Whipped Cream Topping**	Heavy Whipping Cream	82.64		Producers, Fresno, CA, USA
	Granulated Sugar	16.53		Sysco Corp., Houston, TX, USA
	Vanilla Extract	0.83		Cook Flavoring Co., Paso Robles, CA, USA
**Berry Sauce**	Frozen Berry Blend (Raspberries, Blueberries, Blackberries)	64.10		Kirkland Signature, Costco Wholesale Co., Seattle, WA, USA
	Granulated Sugar	22.91		Sysco Corp., Houston, TX, USA
	Water	12.99		-
